# Correction: Hypomethylation mediates genetic association with the major histocompatibility complex genes in Sjögren’s syndrome

**DOI:** 10.1371/journal.pone.0255549

**Published:** 2021-07-27

**Authors:** Calvin Chi, Kimberly E. Taylor, Hong Quach, Diana Quach, Lindsey A. Criswell, Lisa F. Barcellos

## Notice of republication

This article was republished on July 19, 2021, to remove a Supporting Information file that was incorrectly included in the originally published article. Please download this article again to view the correct version.

## References

[1] Chi C, Taylor KE, Quach H, Quach D, Criswell LA, Barcellos LF (2021) Hypomethylation mediates genetic association with the major histocompatibility complex genes in Sjögren’s syndrome. PLoS ONE 16(4): e0248429. 10.1371/journal.pone.0248429 33886574 PMC8062105

