# Supplementary figures and images for: Hypomethylation mediates genetic association with the major histocompatibility complex genes in Sjögren’s syndrome
Source: PLoS One. 2021 Apr 22;16(4):e0248429. doi: 10.1371/journal.pone.0248429 (PMC8062105; doi:10.1371/journal.pone.0248429)

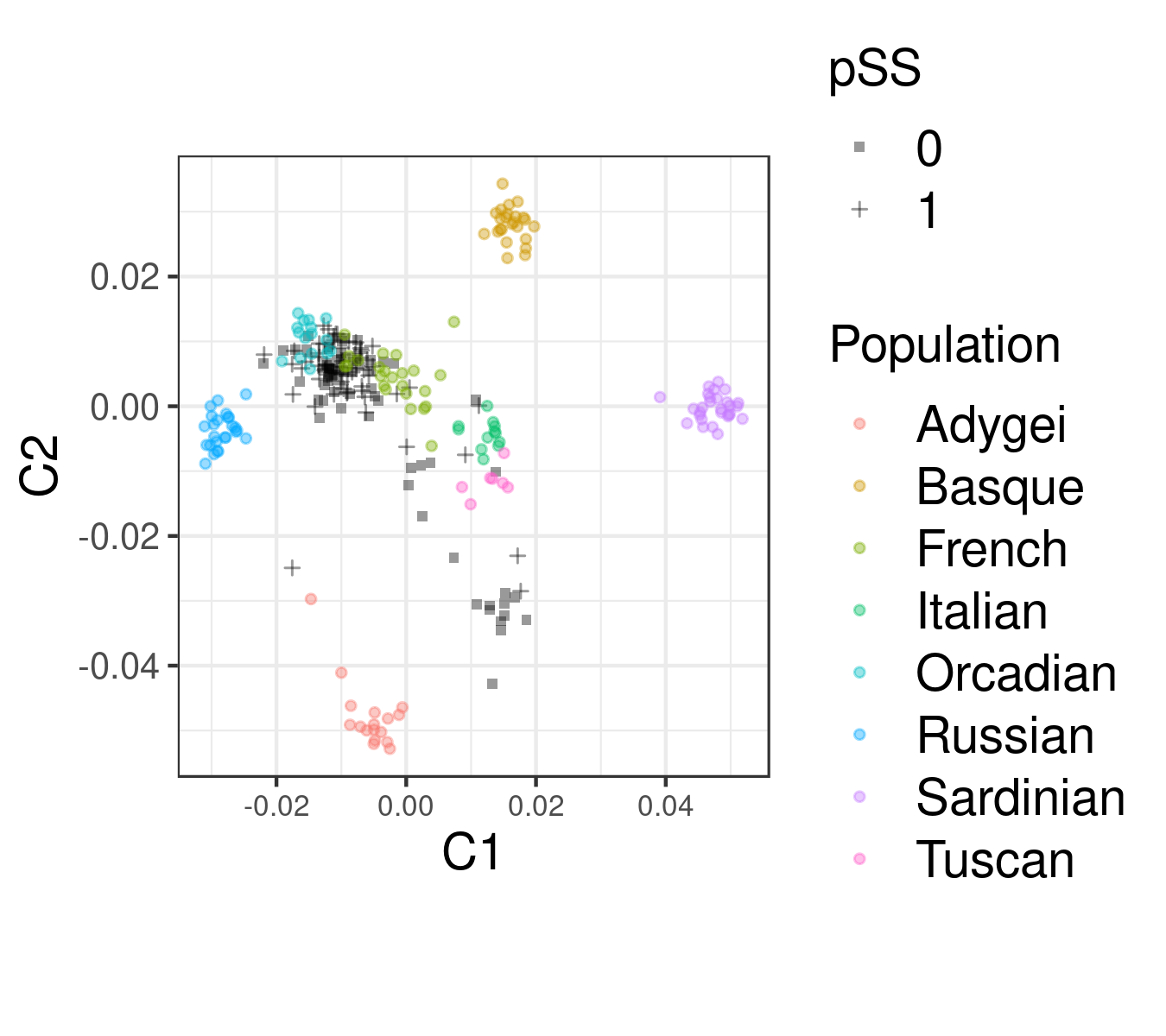

Supplement: S1 Fig — Component 1 (C1) and component 2 (C2) refer to the two dimensions projected to by MDS. (TIFF) [file pone.0248429.s001.tiff]

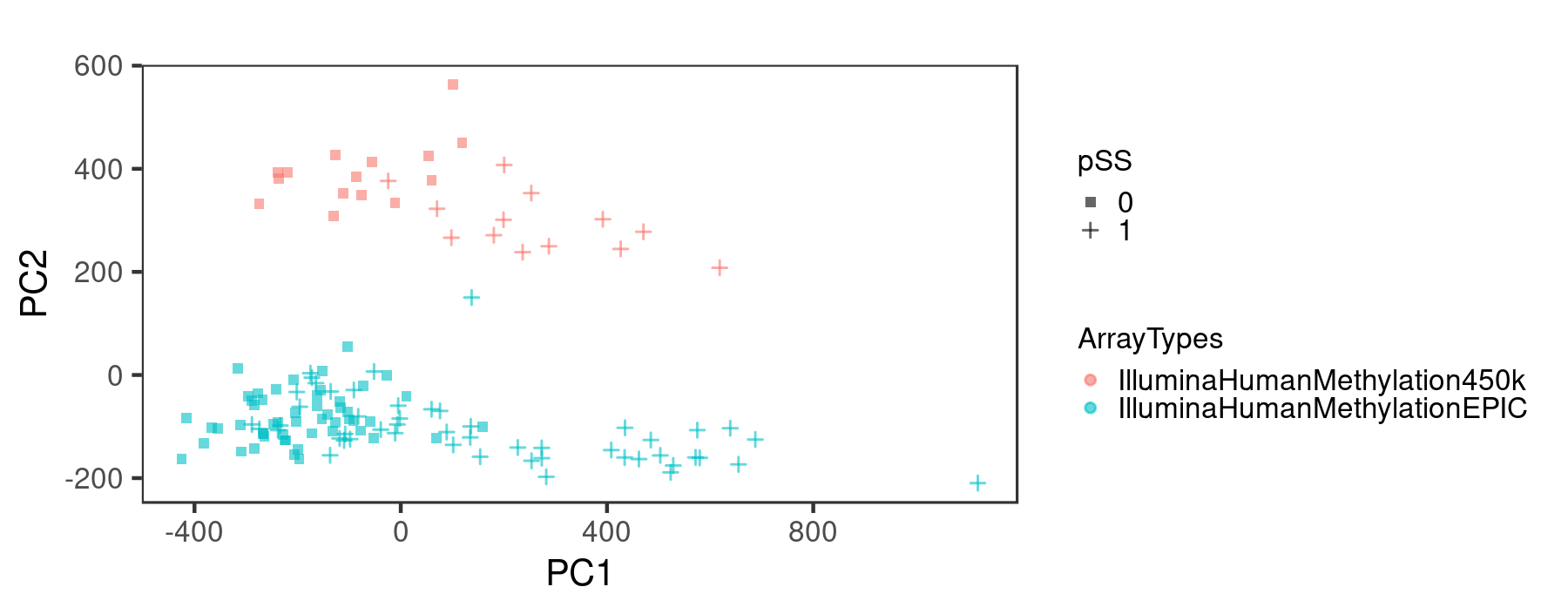

Supplement: S2 Fig — The array type (450K or EPIC) for methylotyping is indicated by color. The array types 450K and EPIC show strong separation on PC2. (TIFF) [file pone.0248429.s002.tiff]

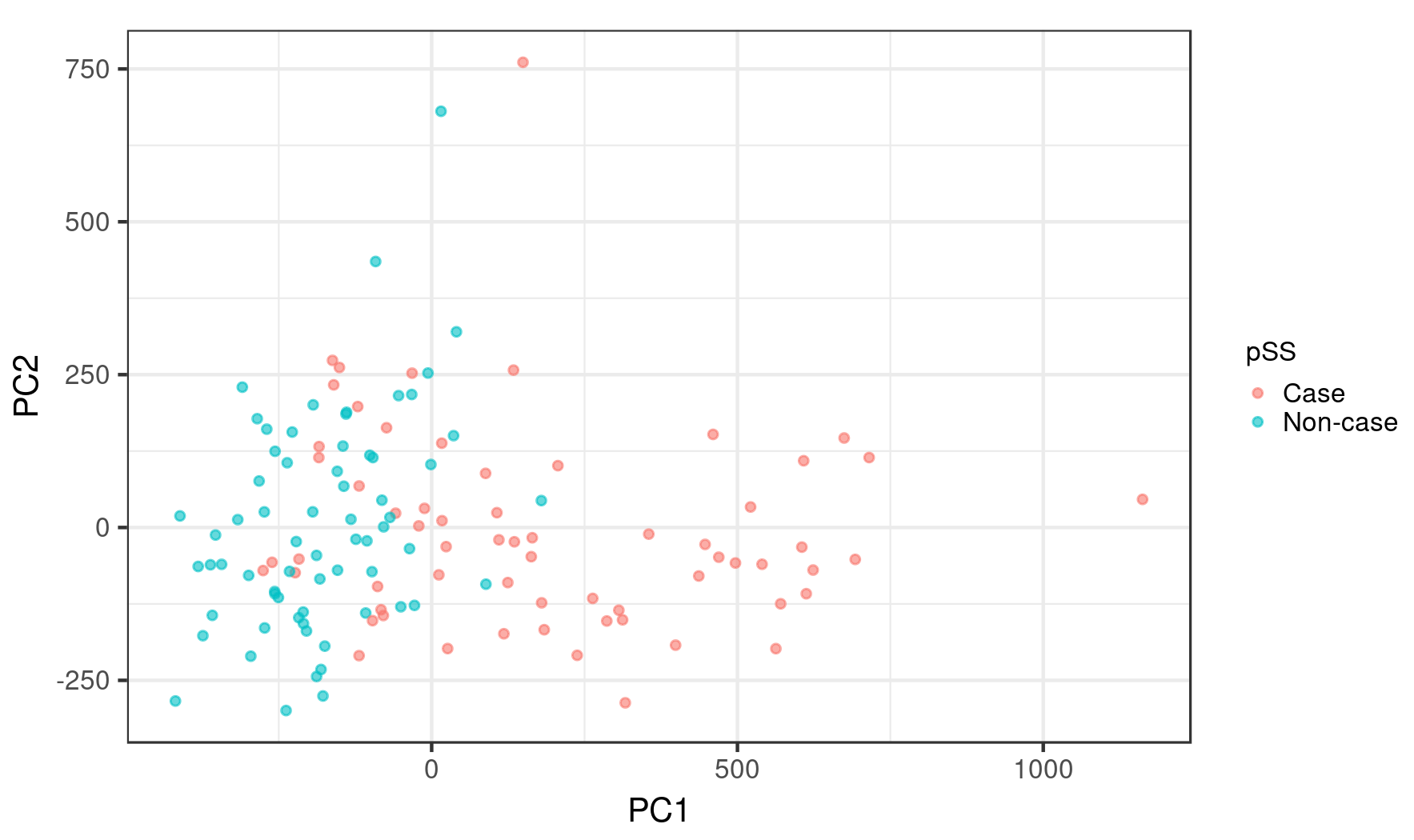

Supplement: S3 Fig — SS case status, as determined by the 2016 ACR/EULAR diagnostic criteria, is indicated by color [32]. Cases and non-cases show strong separation on PC1. (TIFF) [file pone.0248429.s003.tiff]

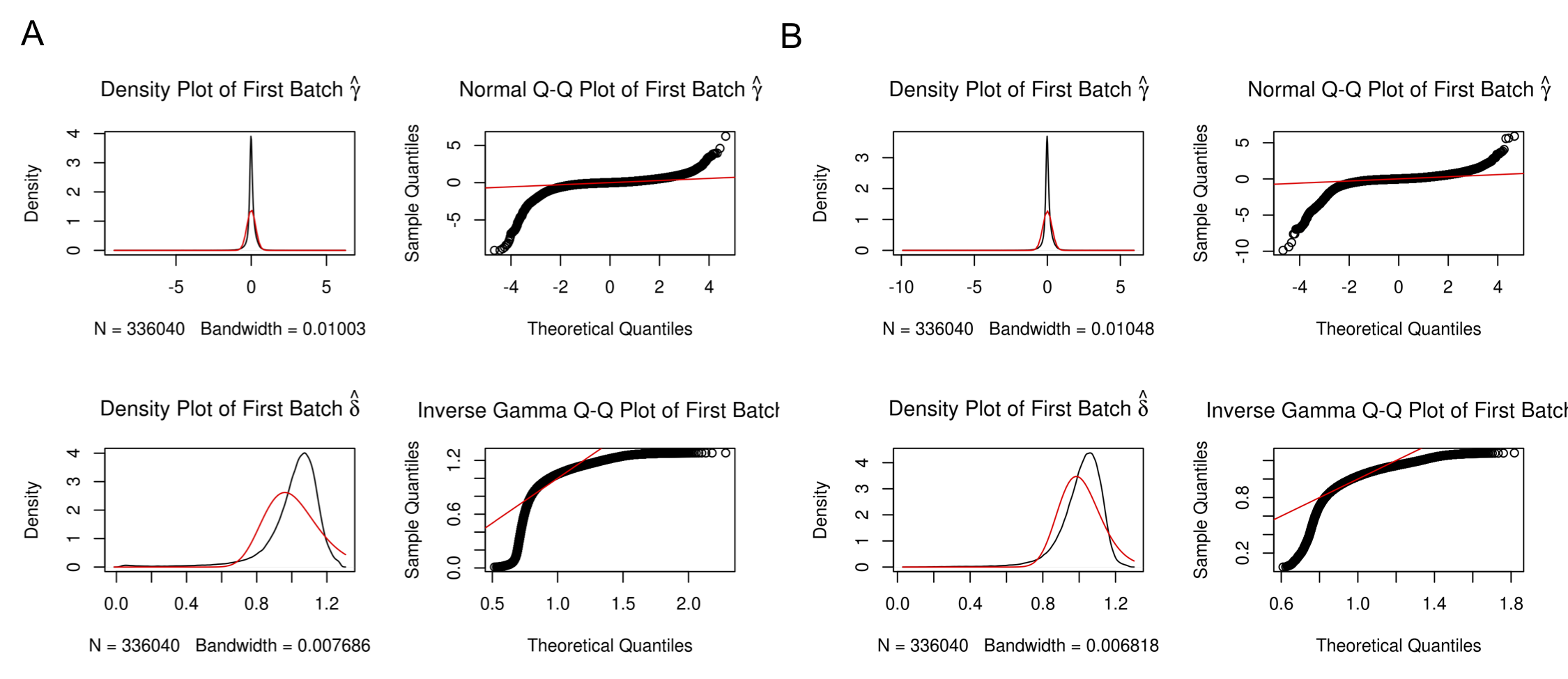

Supplement: S4 Fig — (A) β-values and (B) M-values. (TIFF) [file pone.0248429.s004.tiff]

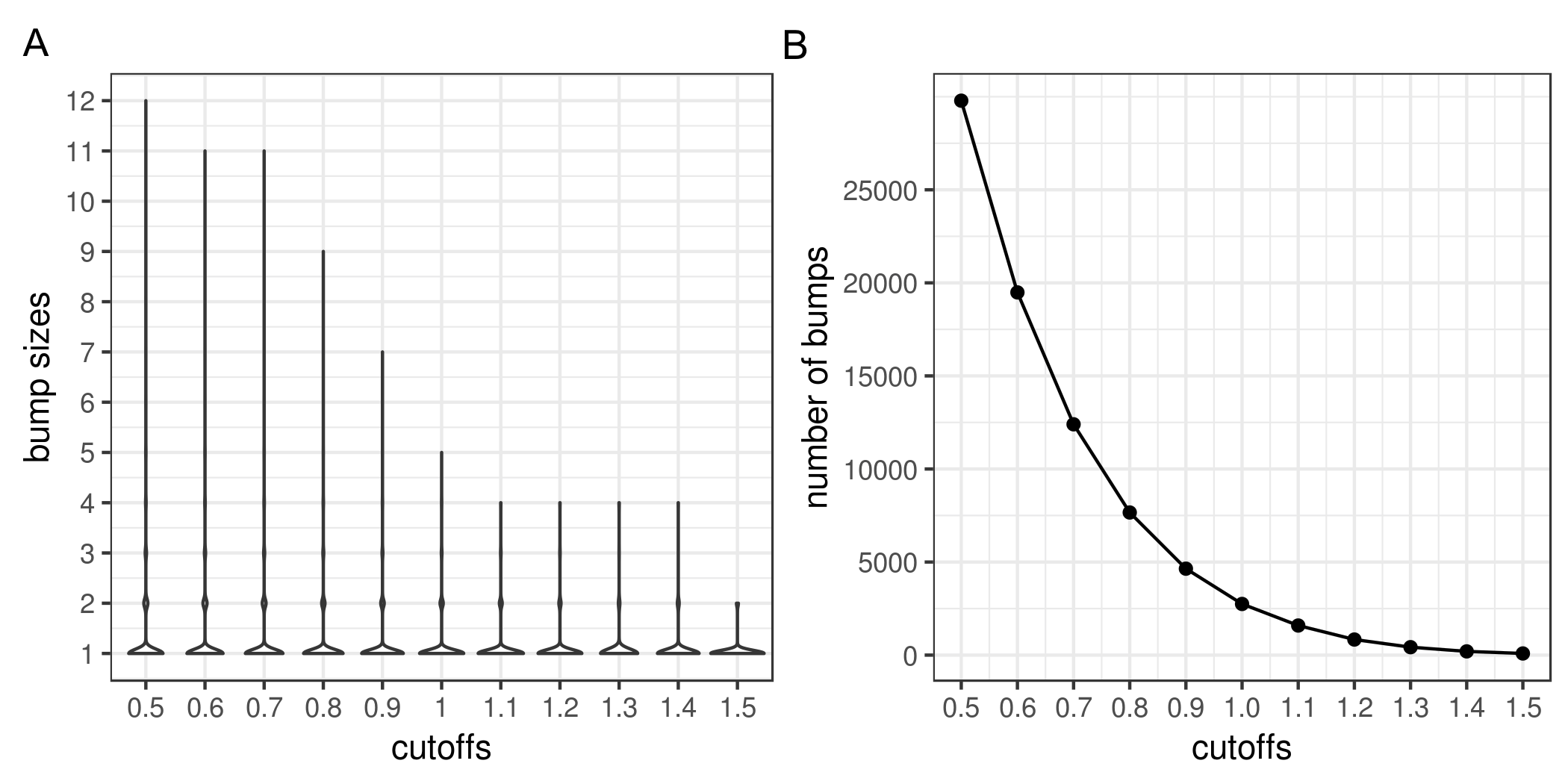

Supplement: S5 Fig — (A) Violin plot of bump sizes at each cutoff (B) Number of bumps discovered at each cutoff. (TIFF) [file pone.0248429.s005.tiff]
