# Supplementary material for: Hypomethylation mediates genetic association with the major histocompatibility complex genes in Sjögren’s syndrome
Source: PLoS One. 2021 Apr 22;16(4):e0248429. doi: 10.1371/journal.pone.0248429 (PMC8062105; doi:10.1371/journal.pone.0248429)
Supplement: S2 Table — (DOCX) [file pone.0248429.s007.docx]

**S2 Table. Gene regions with established or suggestive associations with SS.**

| **Gene** | **Source** |
| --- | --- |
| *STAT4* | Table 2 of Taylor *et al*. and Table 2 of Lessard *et al*. [5,6]. |
| *IRF5* | Table 2 of Taylor *et al*. and Table 2 of Lessard *et al*. [5,6]. |
| *BLK* | Table 2 of Lessard *et al*. [6]. |
| *IL12A* | Table 2 of Lessard *et al*. [6]. |
| *TNIP1* | Table 2 of Lessard *et al*. [6]. |
| *CXCR5* | Table 2 of Lessard *et al*. [6]. |
| *TNFAIP3* | Table 2 of Lessard *et al*. [6]. |
| *DGKQ* | Table 2 of Lessard *et al*. [6]. |
| *ITSN2* | Table 2 of Lessard *et al*. [6]. |
| *HLA-DRA* | Table 1 of Lessard *et al*. [6]. |
| *HLA-DQB1* | Table 2 of Taylor *et al*.,Table 1 of Lessard *et al*., and abstract of Cruz-Tapias *et al*. [5–7]. |
| *HLA-DQA1* | Table 2 of Taylor *et al*.,Table 1 of Lessard *et al*., and abstract of Cruz-Tapias *et al*. [5–7]. |
| *PRCC* | Table 2 of Taylor *et al*.[5]. |
| *SH2D2A* | Table 2 of Taylor *et al*.[5]. |
| *GRIP2* | Table 2 of Taylor *et al*.[5]. |
| *CCDC174* | Table 2 of Taylor *et al*.[5]. |
| *PDE8B* | Table 2 of Taylor *et al*.[5]. |
| *HLA-DRB1* | Table 2 of Taylor *et al*.[5]. |
| *HLA-DQA2* | Table 2 of Taylor *et al*.[5]. |
| *TNPO3* | Table 2 of Taylor *et al*.[5]. |
| *RELN* | Table 2 of Taylor *et al*.[5]. |
| *NACC2* | Table 2 of Taylor *et al*.[5]. |
| *HTR2A* | Table 2 of Taylor *et al*.[5]. |
| *LINC00562* | Table 2 of Taylor *et al*.[5]. |
| *LOC105370283* | Table 2 of Taylor *et al*.[5]. |
| *PTMAP5* | Table 2 of Taylor *et al*.[5]. |
| *MIS18BP1* | Table 2 of Taylor *et al*.[5]. |
| *LINC00871* | Table 2 of Taylor *et al*.[5]. |
| *NFAT5* | Table 2 of Taylor *et al*.[5]. |
| *SHISA9* | Table 2 of Taylor *et al*.[5]. |

**Supplementary References**

1. Du P, Zhang X, Huang C-C, Jafari N, Kibbe WA, Hou L, et al. Comparison of Beta-value and M-value methods for quantifying methylation levels by microarray analysis. BMC Bioinformatics. 2010 Nov 30;11:587.

2. Cole MB, Quach H, Quach D, Baker A, Taylor KE, Barcellos LF, et al. Epigenetic Signatures of Salivary Gland Inflammation in Sjögren’s Syndrome. Arthritis Rheumatol (Hoboken, NJ). 2016;68(12):2936–44.

3. Hjelmervik TOR, Petersen K, Jonassen I, Jonsson R, Bolstad AI. Gene expression profiling of minor salivary glands clearly distinguishes primary Sjögren’s syndrome patients from healthy control subjects. Arthritis Rheum. 2005 May;52(5):1534–44.

4. Millstein J, Volfson D. Computationally efficient permutation-based confidence interval estimation for tail-area FDR. Front Genet. 2013;4:179.

5. Taylor KE, Wong Q, Levine DM, McHugh C, Laurie C, Doheny K, et al. Genome-Wide Association Analysis Reveals Genetic Heterogeneity of Sjögren’s Syndrome According to Ancestry. Arthritis Rheumatol (Hoboken, NJ). 2017;69(6):1294–305.

6. Lessard CJ, Li H, Adrianto I, Ice JA, Rasmussen A, Grundahl KM, et al. Variants at multiple loci implicated in both innate and adaptive immune responses are associated with Sjögren’s syndrome. Nat Genet. 2013 Nov;45(11):1284–94.

7. Cruz-Tapias P, Rojas-Villarraga A, Maier-Moore S, Anaya J-M. HLA and Sjögren’s syndrome susceptibility. A meta-analysis of worldwide studies. Autoimmun Rev. 2012 Feb;11(4):281–7.
