# Supplementary material for: Hypomethylation mediates genetic association with the major histocompatibility complex genes in Sjögren’s syndrome
Source: PLoS One. 2021 Apr 22;16(4):e0248429. doi: 10.1371/journal.pone.0248429 (PMC8062105; doi:10.1371/journal.pone.0248429)
Supplement: S1 Text — (DOCX) [file pone.0248429.s010.docx]

**S1 Text**

**Supplementary Methods**

**Methylotyping and preprocessing**

The two primary measures of DNA methylation of each CpG site are β-values and M-values. A β-value is a ratio of the methylated probe intensity to the sum of methylated and unmethylated probe intensities, which ranges from 0 to 1, and reflects the proportion of methylation at a CpG site, and is more interpretable. The M-value can be derived from a β-value as $\log_{2} \frac{}{1-}$, ranges from -∞ to ∞, and has been the ideal measure to use for identifying differentially methylated CpG sites due to less severe heteroscedasticity[1].

**Removing unwanted DNA methylation variation**

Parametric adjustment was used for *ComBat* because the densities of the additive and multiplicative batch parameters were neither highly skewed nor bimodal (S4 Fig). Missing methylation values were mean imputed per CpG site before applying *ComBat*, then missingness restored after adjustment.

**Dimensionality reduction**

Let $n$ and $p$ be the numbers of subjects and CpG sites respectively. Principal component analysis (PCA) was performed on the centered and scaled β-value matrix $X\in\mathbb{R}^{n \times p}$. Missing values were replaced with per CpG site average before PCA. The symmetric matrix $X^{\top}X\in\mathbb{R}^{p \times p}$ has the eigendecomposition $X^{\top}X=V\Sigma V^{\top}$, where $V\in\mathbb{R}^{p \times p}$ is an orthogonal matrix. In PCA, the columns $v_{1},\ldots,v_{p}$ of $V$ specify the optimal orthogonal directions to project samples onto to preserve variability in the data. Principal component *i (*PC*i*) refers to projections of the *n* samples onto $v_{i}$, which we refer to as the *i*-th principal axis. For example, PC1 is computed as PC1 = $Xv_{1}$, which can be seen here as a linear combination of the CpG site methylation levels in $X$. The first principal axis $v_{1}$ thus contains coefficients for each CpG site, with larger magnitudes indicating greater contribution to PC1. We refer to the absolute value of entries in $v_{1}$ as the loadings for PC1, which we analyzed to determine which CpG sites contributed most to PC1.

**Identification of differentially methylated regions**

Bootstrap resampling was run with option $nullMethod=bootstrap$ so that adjustment covariates were controlled for. One of the most important hyperparameters of *bumphunter* is the effect size cutoff for defining a candidate DMR, where effect size is the estimated expected change in methylation from one group to the other. In this study, a cutoff of 1.0 was chosen to achieve a balance between effect size, number of bumps found, and bump sizes in terms of number of CpG sites (S5 Fig). *Minfi* was used to annotate each DMR with its nearest gene in base pairs, location relative to nearest gene, and location relative to nearest CpG island. The DMR location relative to nearest CpG island was set as the majority location of all CpGs that comprise the DMR. Detailed descriptions of each DMR gene were obtained from the National Center for Biotechnology Information.

**Gene set enrichment analysis**

The GO gene sets total 5,917, with 4,436 derived from biological process ontology, 580 derived from cellular component ontology, and 901 derived from molecular function ontology. Additionally, we included two gene sets consisting of genes shown to be differentially methylated or differentially expressed respectively, between SS cases and controls in LSG[2,3]. We eliminated large gene sets numbering more than 100 genes, retaining approximately 76% of gene sets.

**Mediation analysis with causal inference test**

The CIT was run with default settings for pairs of meQTLs and DMRs whose association was determined to be significant after multiple hypothesis testing adjustment. The q-value, or false discovery rate, for a CIT was estimated based on simulating the null CIT outcome by permuting the relevant variable for each statistical test comprising the CIT, which was done with $n.perm = 100$ permutations, as recommended to be sufficient by Millsten *et al.*[4]. The permutations were specified the same across all tests to account for dependencies among the tests.

**Supplementary Results**

**DNA methylation mediates the effect of meQTLs on SS at the MHC**

On average, the DMR 500 kb windows within the MHC (28,477,797 bp - 33,448,354 bp) have a higher SNP density (0.28 SNPs / kb) than that of non-MHC windows (0.19 SNPs / kb). To investigate whether more meQTL discoveries at the MHC can be explained by higher SNP densities, we randomly down-sampled SNPs in each MHC window to match the average SNP density of non-MHC windows. We then applied the same meQTL analyses as before, using the Benjamini-Hochberg adjusted p-value $\leq$ 0.05 criteria to identify meQTL discoveries. The discovery rate of the MHC windows compared to that of non-MHC windows is expressed as the ratio of the MHC to non-MHC discovery rates (similar to relative risk). A ratio of 1 indicates equivalent discovery rates, above 1 indicates increased discovery rate at the MHC, and below 1 indicates decreased discovery rate at the MHC. We can repeat the down-sampling procedure to generate a 95% confidence interval of discovery rate ratios. From this experiment, we obtain a median discovery rate ratio of 90, with a 95% confidence interval of 60 - 135. This result shows strong evidence that the MHC harbors a higher density of meQTLs than non-MHC regions, keeping SNP densities the same.

**Supplementary Tables**

**S3 Table. Gene regions with established or suggestive associations with SS.**

| **Gene** | **Source** |
| --- | --- |
| *STAT4* | Table 2 of Taylor *et al*. and Table 2 of Lessard *et al*. [5,6]. |
| *IRF5* | Table 2 of Taylor *et al*. and Table 2 of Lessard *et al*. [5,6]. |
| *BLK* | Table 2 of Lessard *et al*. [6]. |
| *IL12A* | Table 2 of Lessard *et al*. [6]. |
| *TNIP1* | Table 2 of Lessard *et al*. [6]. |
| *CXCR5* | Table 2 of Lessard *et al*. [6]. |
| *TNFAIP3* | Table 2 of Lessard *et al*. [6]. |
| *DGKQ* | Table 2 of Lessard *et al*. [6]. |
| *ITSN2* | Table 2 of Lessard *et al*. [6]. |
| *HLA-DRA* | Table 1 of Lessard *et al*. [6]. |
| *HLA-DQB1* | Table 2 of Taylor *et al*.,Table 1 of Lessard *et al*., and abstract of Cruz-Tapias *et al*. [5–7]. |
| *HLA-DQA1* | Table 2 of Taylor *et al*.,Table 1 of Lessard *et al*., and abstract of Cruz-Tapias *et al*. [5–7]. |
| *PRCC* | Table 2 of Taylor *et al*.[5]. |
| *SH2D2A* | Table 2 of Taylor *et al*.[5]. |
| *GRIP2* | Table 2 of Taylor *et al*.[5]. |
| *CCDC174* | Table 2 of Taylor *et al*.[5]. |
| *PDE8B* | Table 2 of Taylor *et al*.[5]. |
| *HLA-DRB1* | Table 2 of Taylor *et al*.[5]. |
| *HLA-DQA2* | Table 2 of Taylor *et al*.[5]. |
| *TNPO3* | Table 2 of Taylor *et al*.[5]. |
| *RELN* | Table 2 of Taylor *et al*.[5]. |
| *NACC2* | Table 2 of Taylor *et al*.[5]. |
| *HTR2A* | Table 2 of Taylor *et al*.[5]. |
| *LINC00562* | Table 2 of Taylor *et al*.[5]. |
| *LOC105370283* | Table 2 of Taylor *et al*.[5]. |
| *PTMAP5* | Table 2 of Taylor *et al*.[5]. |
| *MIS18BP1* | Table 2 of Taylor *et al*.[5]. |
| *LINC00871* | Table 2 of Taylor *et al*.[5]. |
| *NFAT5* | Table 2 of Taylor *et al*.[5]. |
| *SHISA9* | Table 2 of Taylor *et al*.[5]. |

**Supplementary References**

1. Du P, Zhang X, Huang C-C, Jafari N, Kibbe WA, Hou L, et al. Comparison of Beta-value and M-value methods for quantifying methylation levels by microarray analysis. BMC Bioinformatics. 2010 Nov 30;11:587.

2. Cole MB, Quach H, Quach D, Baker A, Taylor KE, Barcellos LF, et al. Epigenetic Signatures of Salivary Gland Inflammation in Sjögren’s Syndrome. Arthritis Rheumatol (Hoboken, NJ). 2016;68(12):2936–44.

3. Hjelmervik TOR, Petersen K, Jonassen I, Jonsson R, Bolstad AI. Gene expression profiling of minor salivary glands clearly distinguishes primary Sjögren’s syndrome patients from healthy control subjects. Arthritis Rheum. 2005 May;52(5):1534–44.

4. Millstein J, Volfson D. Computationally efficient permutation-based confidence interval estimation for tail-area FDR. Front Genet. 2013;4:179.

5. Taylor KE, Wong Q, Levine DM, McHugh C, Laurie C, Doheny K, et al. Genome-Wide Association Analysis Reveals Genetic Heterogeneity of Sjögren’s Syndrome According to Ancestry. Arthritis Rheumatol (Hoboken, NJ). 2017;69(6):1294–305.

6. Lessard CJ, Li H, Adrianto I, Ice JA, Rasmussen A, Grundahl KM, et al. Variants at multiple loci implicated in both innate and adaptive immune responses are associated with Sjögren’s syndrome. Nat Genet. 2013 Nov;45(11):1284–94.

7. Cruz-Tapias P, Rojas-Villarraga A, Maier-Moore S, Anaya J-M. HLA and Sjögren’s syndrome susceptibility. A meta-analysis of worldwide studies. Autoimmun Rev. 2012 Feb;11(4):281–7.
