# Supplementary material for: Hypomethylation mediates genetic association with the major histocompatibility complex genes in Sjögren’s syndrome
Source: PLoS One. 2021 Apr 22;16(4):e0248429. doi: 10.1371/journal.pone.0248429 (PMC8062105; doi:10.1371/journal.pone.0248429)
Supplement: Attachment — Submitted filename: plos_one_response_letter.pdf [file pone.0248429.s011.pdf]

# Reviewer 1

**1) While the first part of the results and Figure 2 tentatively validate the use of symptomatic non-cases as controls, their use is problematic particularly given the lack of information about possible secondary SS in patient diagnosis. Ideally, there should be an age- and ethnicity-matched healthy control group. However, the limited availability of clinical material, especially given the patient cohort sizes in the present study might understandably prevent that. At the very least, information about possible secondary SS should be included or samples with confirmed or suspected SLE or RA should be excluded if this is not possible or available. Case and non-case statistics (e.g. as in Cole et al. 2016 Table 1, reference 12) would also be helpful in this case.**

The proportion of physician confirmed SLE and RA cases and non-cases are now included in the newly included Table 1. These proportions are small (maximum 6%), and are comparable between cases and non-cases (p-value > 0.05). Thus, cases of secondary SS are a small minority and fairly distributed between cases and non-cases in our study, and should not significantly influence the main results. SLE and RA data are included in S1 Table. The newly included Table 1 includes case and non-case statistics of SS phenotypes as well as potential confounders such as age, smoking habit, and drug use.

See subsection “Removing unwanted DNA methylation variation” under the “Materials and Methods” section for details on identifying and addressing potential confounders, such as age and ethnicity. Since genetic ancestry was found to be associated with DNA methylation, we controlled for genetic ancestry in *bumphunter*. We did this by including genotype MDS components C1 - C5 in the *bumphunter* regression (see subsection “Identification of differentially methylated regions” and S1 Figure for MDS plot). This addresses controlling for ethnicity. In our phenotype data (S1 Table), we have available the variables censored age at study visit (age_censored) and self-reported age of SS onset at screening (agess). The distribution of both age variables are not significantly different between cases and non-cases (Table 1), so we did not find it necessary to control for age. For completeness, boxplots of the age variables, by pSS status, are included below:

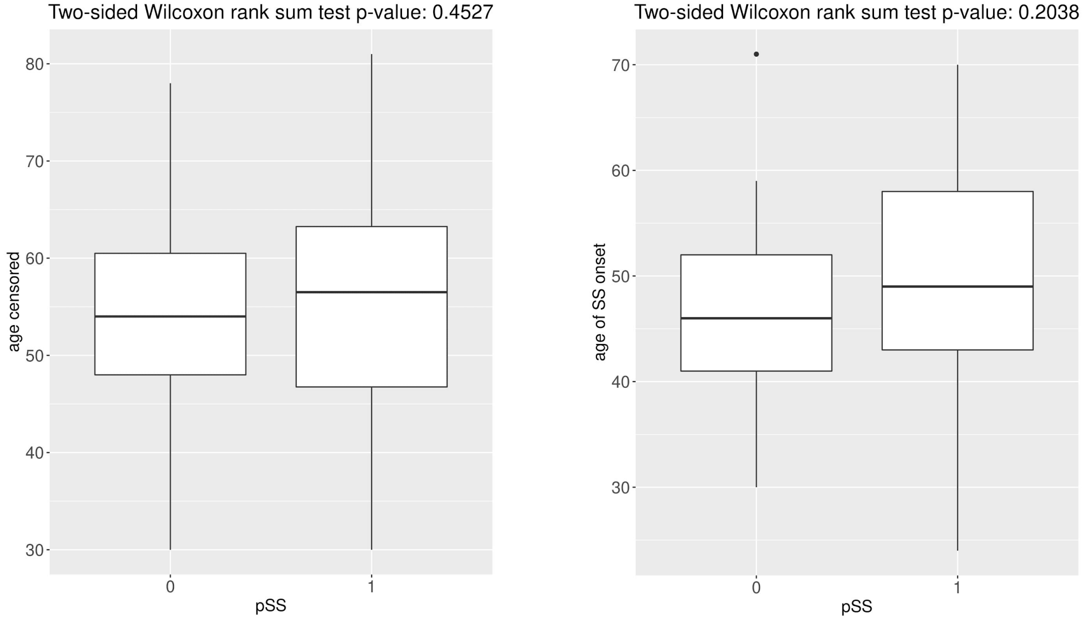

**2) Unless I missed it, the last part of the results (page 15, line 290 onwards, "DNA methylation mediates the effect of meQTLs on SS at the MHC") does not include the use of non-case/control groups. Is it possible to run the CIT algorithm on meQTLs associated to non-case specific DMRs or DMR-SNP candidate pairs on the MHC locus, or to perform an analogous analysis entailing the use of non-cases as a negative control?**

All 131 subjects were used for the mediation analysis, so this includes the non-cases. For example, this involved regressing M-value against number of reference alleles from all 131 subjects. Note that the CIT requires non-cases because without them, the CIT tests cannot evaluate associations involving case status. Just so this is unambiguous, this information is now explicitly stated in the "identification of DNA methylation quantitative trait loci" subsection of the "Materials and Methods" section:

"*The association between a candidate meQTL and DMR was established by regressing the M-value, averaged across CpG sites of the DMR, against genotype encoded as 0, 1, or 2 copies of the reference allele, from all 131 subjects.*"

and in the "Mediation analysis with causal inference test" subsection of the "Materials and Methods" section:

"*The CIT was performed for the identified meQTL-DMR pairs using genotype, DNA methylation, and SS case status from all 131 subjects. The genotype and DNA methylation data are encoded the same way as for the identification of meQTLs.*"

**3) The statement about overlaps of DMRs from Cole et al. 2016 and the present study (page 12, line 265) should be accompanied with a hypergeometric test. Additionally, the last part of the results (page 15) might benefit from hypergeometric tests to further highlight significance, if adequate.**

The differentially methylated genes from Cole *et al.* 2016 is indeed one of the gene sets for which we test for enrichment. This gene set is named "SS DMP genes", and is the most significantly enriched gene set by our hypomethylated genes according to the hypergeometric test (Table 2). The following section is added to make sure we communicate that the significance of the overlap with differentially methylated genes from Cole *et al*. 2016 has been tested**.**

"*Of the 131 individuals in our study, 26 were in a previous LSG study by Cole et al., which identified 57 genes whose promoters were hypomethylated in SS relative to controls. From GSEA, these 57 genes (SS DMP genes) form the top enriched gene set with an adjusted p-value of 1.71E-4 (Table 2).*"

**4) Have the authors accounted for SNP density, which is higher at MHC genes and could result in DMRs being associated to SNPs by chance? Does selecting SNPs in SNP-dense regions at random result in similar results?**

There is no evidence to suggest associations between DMRs with SNPs at the MHC can be explained by a higher density of SNPs. To start, our meQTL discoveries are based on SNPs selected to be in approximate linkage equilibrium based on the correlation coefficient $r^2$ (see subsection "Identification of DNA methylation quantitative trait loci" under "Materials and Methods" for details). This mitigates the effect of multiple discoveries at the MHC due to linkage disequilibrium.

To investigate whether more meQTL discoveries at the MHC can be explained by a higher density of SNPs, we randomly down-sampled SNPs in each DMR 500 kb window (from 250 kb either side of DMR) within the MHC (28,477,797 bp - 33,448,354 bp) to match the average SNP density of windows outside the MHC. Indeed, MHC windows have a density of 0.28 SNPs / kb while non-MHC windows have a density of 0.19 SNPs / kb, leading to about 45 more SNPs tested at MHC windows on average. We then applied the same analyses to identify meQTLs as before, using the Benjamini-Hochberg adjusted p-value <= 0.05 criteria to identify meQTL discoveries. The discovery rate of the MHC windows compared to that of non-MHC windows is expressed as the ratio of the MHC to non-MHC discovery rates (similar to relative risk). A ratio of 1 indicates equivalent discovery rates, above 1 indicates increased discovery rate at the MHC, and below 1 indicates decreased discovery rate at the MHC. Since average densities between MHC and non-MHC windows are now the same, any difference in meQTL discovery rates can be attributed to whether the genomic location is in the MHC or not. We can repeat the down-sampling procedure to generate a 95% confidence interval of discovery rate ratios. From this experiment, we obtain a median discovery rate ratio of 90, with a 95% confidence interval of 60 - 135. A box plot of the discovery rate ratios is shown below.

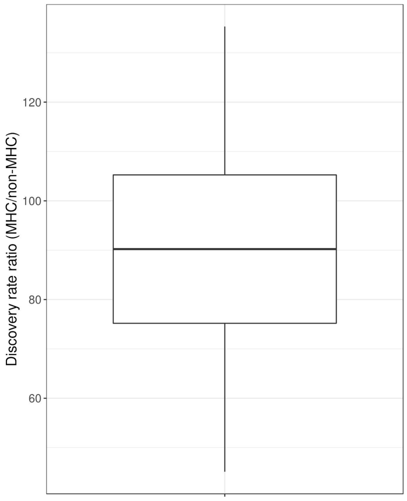


This result shows strong evidence that the MHC harbors a higher density of meQTLs compared to non-MHC regions, keeping SNP densities the same. This additional result is mentioned under

the subsection “DNA methylation mediates the effect of meQTLs on SS at the MHC” of the Results section.

“*Down-sampling SNPs at the MHC to achieve comparable SNP densities to that of non-MHC regions still resulted in a higher meQTL discovery rate at the MHC relative to non-MHC regions (see Supplementary Results for more details). Thus, the higher discovery rate at the MHC cannot be explained by higher SNP densities.*”

**1) The presentation needs to be addressed. In particular, Figure 1 is not described in the text (it should be either in the methods or results), Fig 3C is referenced before 3A in different paragraphs, Fig 4C is referenced before 4A, Fig 4B is not referenced; Figs S1 and S3 are swapped, Figs S2 and S4 are swapped; Tables S2 and S5 are swapped, Tables S3 and S4 are swapped. The quality of the figures should also be worked on**

Figure 1 was previously referenced in the subsection “Mediation analysis with causal inference test” under “Materials and Methods” to help the reader understand the causal mediation model. However, based on reviewer recommendation, this figure is now removed because it is too simple and is not necessary for understanding the causal mediation model described.

All figures submitted satisfy quality requirements by the Preflight Analysis and Conversion Engine digital diagnostic tool, as required by PLOS ONE prior to submission. However, the authors noticed that the figures displayed in the final PDF document compiled by PLOS ONE is noticeably of lower quality than actual figure quality. To view figures in their original quality, the authors recommend downloading the figure image using the download link on the upper right hand corner of the page.

However, the quality of panel A of Figure 4 could indeed be improved. Specifically, Figure 4A shows two UCSC genome browser tracks, one being the zoomed in version of the other. To ensure the texts in the genome browser tracks are visible, we modified Figure 4A to only include the zoomed in genome browser tracks, which is the more important panel of the two. In this revision, Fig 4 is renamed Fig 2, and Fig4A is renamed Fig4C.

The naming of the figures and tables have been corrected. Figure panels are referenced in the order they appear, and each figure panel is now referenced in the text.

**2) It is unclear in the current manuscript what conclusions are to be drawn from the included ancestry information (Fig S2). This should either be expanded or removed altogether.**

We expand on the purpose of ancestry analysis in this revision. S1 fig is now first referenced in the “Study subjects and clinical evaluation” subsection of “Materials and Methods”, where the purpose is to confirm that subjects are non-Hispanic white as claimed, but also to show that some heterogeneity exists in European ancestry. The latter point explains why European

ancestry is still considered a potential confounder in subsection where we aim to remove unwanted DNA methylation variation. Sentences describing this are:

*“Multidimensional scaling (MDS) of genotype data confirms their non-Hispanic white ancestry and suggests that the majority of individuals have either French or Orcadian ancestry (S1 Fig). However, heterogeneity of European ancestry exists in our study subjects.”*

In the subsection “Removing unwanted DNA methylation variation” of Materials and Methods, we explain using the MDS components (analogous to principal components of PCA) to test for association between European ancestry and case status.

Finally, the caption of S1 fig now makes it clear that this is a MDS plot of genotype data.

**3) The submitted data does not seem to be readily available**

- Clinical-related data are provided in S1 Table.
- The genotype data used in this study is the same as the one used in Taylor *et al.*[8]. It has already been deposited in dbGaP, with study accession number phs000672.v1.p1.
- The metadata, non-normalized data, and processed data of DNA methylation has been uploaded to GEO with accession number GSE166373, and will be made publicly available if this work is published.

**4) Can the authors comment on the choice of a 250 kb window rather than 50 kb for SNPs from Smith et al 2014 (ref 37)?**

The choice of a window defined by $\pm 250$ kb on either side of a DMR follows the choice from previous work by Wagner *et al*. [1]. A sample of meQTL papers shows that window size tends to grow with sample size [1-5], probably because more samples are required with more statistical tests in order to control either the false discovery rate or family wise error rate. A table summarizing window and sample sizes for these papers is provided below. There does not appear to be a biologically-justified consensus on window size for meQTL discovery.

| **Paper** | **Window** | **Sample Size(s)** |
|---|---|---|
| Smith *et al.* [2] | $\pm$50 kb | 87 - 106 |
| Bell et al. [3] | $\pm$50 kb | 77 |
| Imgenberg-Kreuz et al. [4] | $\pm$100 kb | 382 |
| Wagner *et al.* [1] | $\pm$250 kb | 62 |
| McRae *et al.* [5] | $\pm$2 mb | 614, 1366 |

With our sample size of 131, we found a ±250 kb window to be one of the largest windows used in studies with comparable sample sizes. Studies using window sizes of at least 1 mb (1,000 kb) tend to involve a few hundred or even more than a thousand samples. In light of the p-values in our study (Table 4 and S4 Table), the number of tests we chose to test (which depends on window size) seems appropriate because many of our significant p-values would not have made the cutoff used in larger studies [5].

Although *cis*-meQTL effects spanning a few mbp have been observed at the MHC [6], McRae *et al.* reported most of the significant *cis*-meQTLs in their work are located within 100 kb of target CpGs [5]. In our study, the average distance between meQTLs and their target DMRs is 153 kb (Fig 2B). Thus, even if our study is adequately powered to detect longer-range *cis*-meQTLs (a determination which also depends on effect size), we do not expect many such *cis*-meQTLs to be missed. We now discuss this in the Introduction and the subsection "Identification of DNA methylation quantitative trait loci" under Materials and Methods, to clarify that all meQTLs reported in this study should be considered short-range *cis*-meQTLs.

**There should be a sentence about DMR identification in the first part of the results (Page 11, line 232-233)**

Following reviewer 2's comments that the PC loading analysis is distracting, the PC loading analysis has been removed. Thus, a short description of DMR identification no longer needs to be provided. Instead, the first subsection under Results is focused on characterizing the study cohort in terms of SS phenotypes, global DNA methylation patterns, potential confounders, and co-morbidities.

**In Fig S2, what are C1 and C2?**

S2 Fig is now re-named as S1 Fig. The added figure caption - "Component 1 (C1) and component 2 (C2) refer to the two dimensions projected to by MDS." explains what C1 and C2 are in the figure.

# Reviewer 2

**What is the purpose of the 1st paragraph? In line 233 the authors reference DMRs that are introduced and estimated in the next section. The sentence "We observed that 233 CpG sites in DMRs significantly contributed to PC1 on average, with an average absolute 234 loading percentile of 94%" is not of importance. On the contrary, if one observes skewness of the PC loadings it is usually an indication of non-normal behavior of the PCs. Generally, the overall premise of the paragraph/PCA analysis is distracting. The objective of this paper is to identify DMRs and any possible mediation of the SS genetics (? See comment below). What the average reader expects in this first paragraph is an introduction of the cohort and the data.**

The original purpose of the first paragraph is to highlight the strong relevance of DNA methylation in the context of SS and LSG. However, we agree that the technical analysis of CpG site principal component loadings is not necessary for making this point, and can be distracting for the average reader. Hence, we removed the PC loadings analysis in this revision.

Following reviewer recommendation, we instead devote the first subsection of the Results section to characterizing the study cohort. Specifically we discuss the clinical and global DNA methylation differences between cases and non-cases. This establishes that (1) cases and non-cases have very different SS phenotypes even though non-cases have SS-related phenotypes, and (2) to confirm in this larger LSG study that DNA methylation analysis is worth studying in the context of SS. Additionally, this subsection also establishes that our main results are unlikely to be significantly influenced by available potential confounders and co-morbidities (Table 1).

**MHC DMRs: the authors did remove probes that overlapped polymorphic positions as part of their QC. This step is usually accomplished leveraging lists provided by various tools, e.g. minfi. Did the authors examine post-hoc whether any of the identified DMRs overlapped with any known variant, especially within the MHC?**

Yes, we did remove probes that overlapped polymorphic positions as part of QC. This is stated in the paper:

"*probes with SNPs with minor allele frequency greater than 0% at either the probe site, CpG interrogation site, or single nucleotide extension were removed*"

Following reviewer recommendation, we examined whether any of the identified DMRs overlapped with genetic variants known for association with SS. Specifically, if the genetic coordinate range (start to end) of a gene overlaps with that of a DMR, then the SNPs in the gene region are considered to overlap with the DMR. The gene set we consider comes from two genome-wide association studies and a meta analysis of HLA association with SS[8,10,11], totaling 30 genes (S3 Table). The DMR set comes directly from the 215 DMRs we identified using *Bumphunter* (S2 Table). From this examination, we found no overlap between our DMRs and genetic variants at established risk and protective loci for SS, even at the MHC.

Although Miceli-Richard *et al*. observed an overlap between genetic risk loci with differentially methylated DNA regions, our study differs in a few key ways[7]. First, while their results are based on the study of T cells and B cells, our results are based on the labial salivary glands (LSGs). Second, in Miceli-Richard *et al*., one differentially methylated CpG site in a gene is sufficient to be considered an overlap[7]. In contrast, our definition of a differentially methylated region requires two contiguous CpG sites to be differentially methylated, all in the same direction. It cannot be ruled out based on our analysis that some CpG sites in known genetic risk loci could also be differentially methylated, even though no DMRs overlap with genetic variants at established SS genetic risk/protective loci.

This result and discussion is now included under subsection “Hypomethylation of genes involved in immune response” of Results.

**What is the justification of testing for meQTLs within =-250Kb and not a large region, e.g. +-1MBps? Given the long-range LD within MHC, one would expect this region to be larger for the MHC DMRs.**

The choice of a window defined by $\pm 250$ kb on either side of a DMR follows the choice from previous work by Wagner *et al*. [1]. A sample of meQTL papers shows that window size tends to grow with sample size [1-5], probably because more samples are required with more statistical tests in order to control either the false discovery rate or family wise error rate. A table summarizing window and sample sizes for these papers is provided below. There does not appear to be a biologically-justified consensus on window size for meQTL discovery.

| **Paper** | **Window** | **Sample Size(s)** |
|---|---|---|
| Smith *et al.* [2] | $\pm$50 kb | 87 - 106 |
| Bell et al. [3] | $\pm$50 kb | 77 |
| Imgenberg-Kreuz et al. [4] | $\pm$100 kb | 382 |
| Wagner *et al.* [1] | $\pm$250 kb | 62 |
| McRae *et al.* [5] | $\pm$2 mb | 614, 1366 |

With our sample size of 131, we found a $\pm$250 kb window to be one of the largest windows used in studies with comparable sample sizes. Studies using window sizes of at least 1 mb (1,000 kb) tend to involve a few hundred or even more than a thousand samples. In light of the p-values in our study (Table 4 and S4 Table), the number of tests we chose to test (which depends on window size) seems appropriate because many of our significant p-values would not have made the cutoff used in larger studies[5].

Although *cis*-meQTL effects spanning a few mbp have been observed at the MHC[6], McRae *et al.* reported most of the significant *cis*-meQTLs in their work are located within 100 kb of target CpGs[5]. In our study, the average distance between meQTLs and their target DMRs is 153 kb (Fig 2B). Thus, even if our study is adequately powered to detect longer-range *cis*-meQTLs (a determination which also depends on effect size), we do not expect many such *cis*-meQTLs to be missed. We now discuss this in the Introduction and the subsection “Identification of DNA methylation quantitative trait loci” under Materials and Methods, to clarify that all meQTLs reported in this study should be considered short-range *cis*-meQTLs.

**The authors’ main finding, the one that dictates the title of the paper, is compressed in the last paragraph. It is not easy to identify which are the six MHC variants that are reported in the SS GWAS and what were the reported ORs and p-values. For example,**

**were these associations with the variants or respective HLA alleles? What is the LD of the reported MDR-meQTL with the GWAs hits? This part of the paper comes across as hastily put together although there is room to dig deeper into the reported associations.**

We make the following main changes/additions:

- All meQTLs supporting the causal mediation model were examined for their association with SS (defined by the 2016 ACR/EULAR classification criteria[9]) in previous European GWAS for SS by Taylor *et al*.[8]. These results are listed in Table 5, which includes p-values, odds ratios, and confidence intervals.
- Determine the linkage disequilibrium (LD) patterns among MHC meQTLs (six variants) that support causal mediation model. These meQTLs are in mild LD with each other, as shown in Fig 4A and S5 Table A. Additionally, multivariate logistic regression regressing case status against all six MHC meQTL variants show modest evidence of independence effects.
- Determine the LD patterns between the same set of MHC meQTLs and established genetic risk loci for SS at the MHC in Europeans[8,10]. Figure 4B and S5 table B show that some meQTLs are in stronger LD with different risk alleles in HLA gene regions.

A detailed discussion of these additional analyses is included in the subsection “DNA methylation mediates the effect of meQTLs on SS at the MHC” in the Results section, expanding the portion discussing the MHC meQTLs from one paragraph to three paragraphs, with two additional main text figures (Fig 3 and Fig 4), two additional main text tables (Table 5 and Table 6), and one supplementary table (S5 Table).

**PCA plots: There are generally two PCA plots presented, Figure 2 and Sup Fig 1. Why do these PCA plots look so different? One would expect theses to be identical, given that the same exact data are utilized or should be utilized.**

One PCA plot is based on β-values prior to batch-correction with *ComBat*, and the other is based on β-values after batch-correction with *ComBat*. The title of the caption indicates this for these figures, now renamed S2 Fig and S3 Fig:

- S2 Fig. PCA of processed β-values, prior to batch-correction with *ComBat*.
- S3 Fig. PCA of processed β-values, after batch-correction with *ComBat*.

Indeed, after batch correction for “ArrayType” (EPIC vs 450k), we no longer see association between “ArrayType” with PC2. This is shown in S3 fig. See “Supplementary Methods” for details on *Combat* usage.

**Figure 1: this is a simple representation of the genetic to methylation to phenotype model. It lacks other possible explanations of the causal relationship, e.g. reverse causation or independent associations. It is of little to no value and it should be removed.**

Figure 1 has been removed.

**Figure 2: how many probes were used for the PCA analysis? What do the authors mean by “preprocessed”? Do they imply QC-ed? This plot has a better place in the Supplementary Material rather than the main manuscript.**

A total of 336,040 probes were used for the PCA analysis. By “preprocessed”, we describe the DNA methylation data after the data processing steps of normalization and quality control (QC), the details of which are in the subsection “Methylotyping and data processing” under the Materials and Methods section. Thus, these 336,040 probes have survived QC. To reduce confusion, we renamed “preprocessed” as simply “processed”. To be clear, this does not include batch-correction with *ComBat*, described in the following subsection of “Removing unwanted DNA methylation variation”. The title caption of PCA plots describes whether the PCA is based on methylation data prior or after batch-correction with *ComBat*. The original plot referred to by the reviewer has been moved to Supplementary Material as S3 fig.

**Figure 3: Panel B, could you replace the scientific notation with numbers? Especially the X axis can be represented in Mbps. Panel C, what is conveyed in this plot, especially in the X axis? What is the overall purpose of it? The main things one should review in the PC loadings are i) their distribution, and ii) the top loadings if the distribution is normal.**

The scientific notation of Fig 3B (now Fig 1B) is now replaced with numbers in units of megabase pairs (mb). Following another comment by the reviewer, we removed the PC loadings analysis in this revision, so panel C has been removed from this figure as well.

**Figure 4: Panel A is of extremely low quality and there is not text visible. It cannot be evaluated what is plotted. Panel B is for which probe(s)/DMR? The respective legend does not explain which probe(s) is/are displayed. Panel C, is significance defined at p-value of <=0.05 as the legend suggests? The authors have not discussed the clear bimodal distribution of the “Significant” distribution. What is a possible explanation?**

- Panel A (now Panel C): only the “zoomed in” view of the genome browser track is included in Panel C, which is the most important view. The genome browser track image, which is obtained from the UCSC genome browser, is of the highest resolution possible. The text displays clearly even after zooming in into the image.
- Panel B (now Panel A): the corresponding DMR is “chr6:32810706-32810742”, or DMR at chromosome 6 from genetic positions 32,810,706 to 32,810,742. This is included in the legend for panel B. The CpG sites of each DMR can be found in S2 Table B. In this case, the CpG sites are cg24898914 and cg00533183.
- Panel C (now Panel B) The significance is defined at Benjamini-Hochberg adjusted p-value <= 0.05, so the density plot for this group is based on distances of the 26 meQTL-DMR pairs described in the main text. This criteria has been added to the figure caption for clarity. Due to small sample size and smoothing for the density plot, the smaller of the two peaks is very likely an artifact, and the large peak is not. Indeed, from the distance histogram below of meQTLs satisfying Benjamini-Hochberg adjusted p-value <= 0.05, the smaller peak of the density plot is due to the three meQTLs at

approximately 75 kb from their corresponding DMR. Most of the distribution mass is still placed around 150 kb, and is responsible for the larger peak observed. This explanation of the the "bimodal distribution" had been added to the main text - "*While the density plot of the meQTL-DMR distances appears somewhat bimodal, the smaller peak at around 60 kb is most likely an artifact due to small sample size and the smoothing process of a density plot. From S4 Table, there are only 3 meQTL-DMR distances ranging from 75 kb to 80 kb.*"

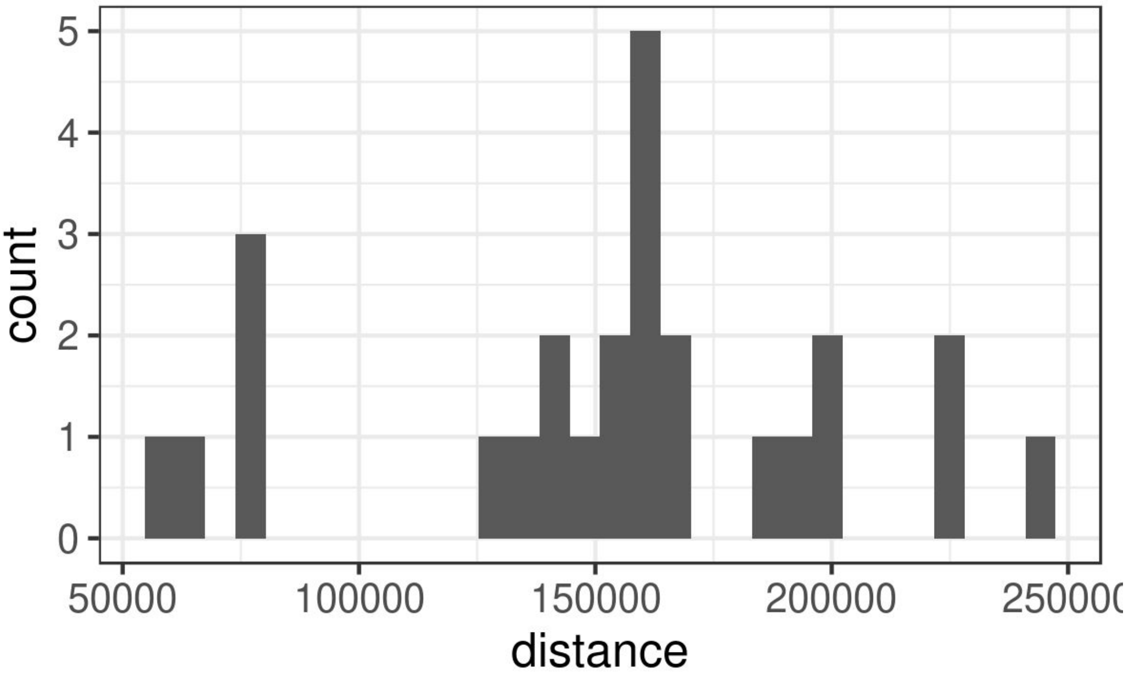


**Supplementary Tables: Most seem to be mislabeled, e.g. Sup Table 4 is actually Sup. Table 5. More information is needed to explain what is displayed in each of the tables.**

The tables are now labeled correctly. The data contained in all supplementary tables are explained at the end of the main text in the form of table titles and captions, following PLOS ONE instructions - "List supporting information captions at the end of the manuscript file. Do not submit captions in a separate file." Note S3 Table is listed in the supplementary text to easily provide citations.

## References

1. Wagner, James R., et al. "The relationship between DNA methylation, genetic and expression inter-individual variation in untransformed human fibroblasts." *Genome biology* 15.2 (2014): R37.
2. Smith, Alicia K., et al. "Methylation quantitative trait loci (meQTLs) are consistently detected across ancestry, developmental stage, and tissue type." BMC genomics 15.1 (2014): 145.
3. Bell, Jordana T., et al. "DNA methylation patterns associate with genetic and gene expression variation in HapMap cell lines." *Genome biology* 12.1 (2011): R10.

4. Imgenberg-Kreuz, Juliana, et al. "Genome-wide DNA methylation analysis in multiple tissues in primary Sjögren's syndrome reveals regulatory effects at interferon-induced genes." Annals of the rheumatic diseases 75.11 (2016): 2029-2036.
5. McRae, Allan F., et al. "Identification of 55,000 replicated DNA methylation QTL." Scientific reports 8.1 (2018): 1-9.
6. McRae, Allan F., et al. "Contribution of genetic variation to transgenerational inheritance of DNA methylation." *Genome biology* 15.5 (2014): R73.
7. Miceli-Richard, Corinne, et al. "Overlap between differentially methylated DNA regions in blood B lymphocytes and genetic at-risk loci in primary Sjögren's syndrome." Annals of the rheumatic diseases 75.5 (2016): 933-940.
8. Taylor, Kimberly E., et al. "Genome-wide association analysis reveals genetic heterogeneity of Sjögren's syndrome according to ancestry." *Arthritis & Rheumatology* 69.6 (2017): 1294-1305.
9. Shiboski, Caroline H., et al. "2016 American College of Rheumatology/European League Against Rheumatism classification criteria for primary Sjögren's syndrome: a consensus and data-driven methodology involving three international patient cohorts." *Annals of the rheumatic diseases* 76.1 (2017): 9-16.
10. Lessard, Christopher J., et al. "Variants at multiple loci implicated in both innate and adaptive immune responses are associated with Sjögren's syndrome." *Nature genetics* 45.11 (2013): 1284-1292.
11. Cruz-Tapias, Paola, et al. "HLA and Sjögren's syndrome susceptibility. A meta-analysis of worldwide studies." Autoimmunity reviews 11.4 (2012): 281-287.
